# Unveiling the therapeutic mechanisms of Saorilao-4 decoction in pulmonary fibrosis through metabolomics and transcriptomics

**DOI:** 10.3389/fnut.2025.1612149

**Published:** 2025-08-11

**Authors:** Jiali Liu, Xiaowen Song, Xinni Song, Xinyue Fu, Shufang Niu, Hong Chang, Songli Shi, Meiqing Yang, Ruiqi Zhao, Peng Wang, Jun Qi, Wanfu Bai

**Affiliations:** ^1^Department of Pharmacy, Baotou Medical College, Baotou, China; ^2^The Second Affiliated Hospital of Baotou Medical College, Baotou, China; ^3^The First Affiliated Hospital of Baotou Medical College, Baotou, China; ^4^Baotou Medical College, Institute of Bioactive Substance and Function of Mongolian Medicine and Chinese Materia Medica, Baotou, China

**Keywords:** pulmonary fibrosis, Saorilao-4 decoction, mongolian medicine, transcriptomics, metabolomics

## Abstract

Pulmonary fibrosis (PF) is a challenging and intricate lung ailment to treat. Saorilao-4 decoction (SRL) is a traditional Mongolian medicine remedy frequently utilized in the management of lung disorders. This study employed transcriptomics and metabolomics to explore the molecular mechanisms of SRL in the bleomycin-induced PF rat model. Additionally, histopathological analysis and the assessment of serum biochemical indices were utilized to investigate the potential mitigating effects of SRL on PF. Transcriptomics and metabolomics analyses indicate that SRL primarily modulates the adenosine monophosphate-activated protein kinase (AMPK) and peroxisome proliferator-activated receptors (PPAR) signaling pathways by regulating genes such as *Scd*, *Fads2*, *Cpt1a*, *Lipe*, *Pfkfb3*, and *Hmgcs2*. Thereby influencing biomarkers such as S-Adenosylmethioninamine (SAM), 16-hydroxyhexadecanoic acid, and proline, mitigating metabolic disorders in pathways such as arginine and proline metabolism, vitamin B6 metabolism, fatty acid degradation, and arachidonic acid metabolism. The present study reveals the regulation of unsaturated fatty acid-related metabolic pathways *in vivo* and elucidates the mechanism of action of SRL in ameliorating PF.

## Graphical abstract

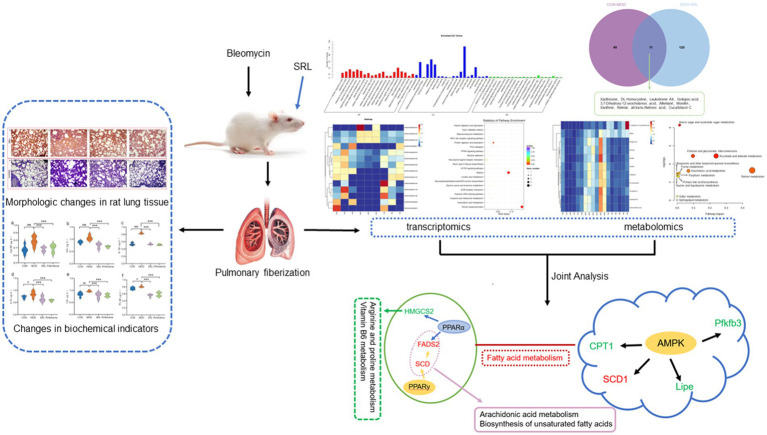

## 1 Introduction

Pulmonary fibrosis (PF) is a chronic and progressive lung disease (1), It is characterized by chronic inflammation, characterized by the release of cytokines such as transforming growth factor beta (TGF-*β*) and interleukin 6 (IL-6), triggered by repeated alveolar damage. Fibrosis results from excessive deposition of extracellular matrix (ECM) components, such as collagen types I and III, by activated fibroblasts. Structural damage occurs due to the destruction of alveolar epithelium and thickening of the lung interstitium (2). Pulmonary fibrosis ultimately affects gas exchange. Common symptoms include escalating dyspnea, non-productive cough, thoracic discomfort, fatigue, and weight loss, ultimately leading to respiratory insufficiency (3). Although various factors such as environmental exposures, medication reactions, autoimmune disorders, and genetic susceptibilities are implicated in the pathogenesis of PF (4), the exact mechanisms underlying the disease remain incompletely elucidated. In the realm of clinical therapy, pirfenidone functions to impede the proliferation and functionality of fibroblasts, thereby diminishing the synthesis of fibrotic proteins and the buildup of extracellular matrix (5). Similarly, nintedanib slows down the process of fibrosis by blocking the signaling of fibroblast growth factors, thereby hindering fibroblast proliferation, migration, and differentiation (6). Nevertheless, both pharmaceutical agents are linked to notable hepatic dysfunction and renal toxicity, posing challenges to sustaining prolonged patient compliance (7, 8). Traditional Mongolian medicine is undergoing modernization driven by advances in medical technology. By integrating metabolomics, network pharmacology, and other biotechnological methods, researchers have significantly improved the scientific understanding of its treatments, including the elucidation of concepts such as the multi-target mechanism of action of the three-flavor *Salvia miltiorrhiza* formula. With the advantages of low drug resistance, high safety, and minimal side effects, traditional Mongolian medicine has demonstrated significant efficacy and broad development prospects in the clinical treatment of pulmonary diseases and other fields (9).

The Saorilao-4 decoction (SRL), a traditional Mongolian herbal concoction containing Glehnia littoralis Fr. Schmidt ex Miq., Polygonum bistorta L., *Glycyrrhiza uralensis* Fisch., and Laccifer lacca Kerr (Lacciferidae Cockerell), is renowned (10) for its effectiveness in alleviating heat, cough, and phlegm (11). In Mongolia, clinical practice predominantly employs this formulation for treating symptoms such as cough and sharp chest and back pain (12), showcasing notable therapeutic benefits. Previous research conducted by our team has demonstrated that the use of SRL can effectively ameliorate lung pathology in a PF rat model, potentially halting or reversing the progression of the disease. Pre-pharmacodynamic studies conducted by our group found that SRL was able to alleviate PF disease, and that the low-dose group of SRL (0.899 g/kg) was the best administered dose group (13). The mechanism of action of SRL may involve the regulation of the Plcd3-OT1/rno-miR-150-3p/Fkbp5 axis, resulting in a significant decrease in long non-coding RNAs–cyclooxygenase 2 (LncRNA-COX2) expression, leading to a decrease in EGR1 expression and an increase in miR-150-3p levels. These findings suggest that SRL exhibits anti-fibrotic properties and may have a significant impact on the development of PF (14). Overall, the results of the study indicate that SRL could be a promising therapeutic option for the treatment of PF.

However, due to the multi-component, multi-target therapeutic modality of SRL treatment, complex interactions may occur among various components, leading to the current lack of clarity regarding the specific molecular mechanisms by which SRL regulates within the body. Thus, there is a pressing need for a systematic investigation of SRL’s efficacy in treating PF. Recent advancements in multi-omics research, facilitated by quantitative and high-throughput screening technologies, have significantly contributed to this field (15, 16). High-throughput technologies in histological studies have allowed us to identify the distribution of abnormal cell states and specific cell populations related to PF. Advancements in high-throughput sequencing and mass spectrometry have revolutionized systems biology and life science research. Multi-omics analyses are widely used in biomedical research, including disease studies and drug development. Integrating various histological data allows researchers to gain a deeper understanding of the molecular basis of diseases and to identify new disease markers and therapeutic targets. These discoveries offer new objectives and directions for future research and therapeutic strategies. Additionally, the accuracy and reproducibility of multi-omics analyses make them powerful for clinical translation. This study utilizes a multi-omics approach for the first time to investigate SRL’s mechanism in treating PF, enhancing understanding of biological processes and disease pathogenesis. The study elucidated the mechanism by which SRL alleviates PF through the integration of transcriptomics and metabolomics methodologies. This research provides a novel perspective for investigating the efficacy and safety profiles of Mongolian medicine compounds.

## 2 Materials and methods

### 2.1 Materials and reagents

The Saorilao-4 decoction was obtained from the Inner Mongolia International Mongolian Medical Hospital, Hohhot City, Inner Mongolia Autonomous Region, China, with batch number 20200414 and a specification of 3 g per bag, containing a mass ratio of 5:3:3:3 of *G. littoralis* Fr. Schmidt ex Miq., *P. bistorta* L., *G. uralensis* Fisch., and Laccifer lacca Kerr (Lacciferidae Cockerell). A decoction was prepared from a fine powder. Quality control was in accordance with the “Drug Standards of the Ministry of Health of the People’s Republic of China Mongolian Medicine.” Bleomycin for injection (batch number: SL30191404, specification 100 mg/mL) was purchased from Beijing Coolaibo Technology Co., Ltd., Beijing. Hematoxylin and eosin (H&E) staining kit (batch number: 20191231) was purchased from Beijing Bioabo Technology Co., Ltd., Beijing. Masson’s trichrome staining solution (batch number 20191025), interleukin-1β (IL-1β) kit (batch number: CK-E30206), IL-6 kit (batch number: CK-E30219), hyaluronic acid (HA) kit (batch number: CK-E30811), laminin (LN) kit (batch number: CK-E30254), type III procollagen (PC-III) kit (batch number: CK-E31310), and type IV collagen (Col-IV) kit (batch number: CK-E34380) were purchased from Nanjing Jianxeng Biological Engineering Institute, Nanjing City, Jiangsu Province. Sodium chloride injection (batch number: 2012056G, specification 200 mL, 1.8 g), used as normal saline, was purchased from Shandong Hualu Pharmaceutical Co., Ltd., Jining City, Shandong Province. Pirfenidone capsules were commercially obtained from Beijing Continent Pharmaceuticals Co., Ltd., Beijing (National Drug Approval Number: H20133376, supplementary batch number: 20241003), an approved manufacturer of this medication in China. Methanol was analytically pure. Distilled water was used.

### 2.2 Animals and drugs

The animal experiments were ethically approved by the Animal Experimental Ethics Committee of Baotou Medical College under protocol 2022–96. All methods are performed in accordance with relevant guidelines and regulations. A total of 48 6–8-week-old healthy adult male SPF-grade Sprague Dawle (SD) rats weighing between 220 and 250 g were obtained from the China Academy of Pharmaceutical Inspection and Research, Beijing with animal production license number SCXK (Beijing) 2017–0005, were housed in controlled conditions at a temperature of 22 ± 2°C and a humidity of 50 ± 10%. Rats were fed a maintenance diet (Keao Xieli Feed Co., Ltd.) and given purified water. In our study, we determined the doses of SRL and pirfenidone according to the dosage specified in the drug instructions and calculated based on body surface area. Our published pharmacodynamic research papers have demonstrated the efficacy and safety of our approach. The SRL formula was dissolved in physiological saline to achieve a crude drug concentration of 0.899 g/kg for administration in the study (13).

### 2.3 PF model preparation and drug administration

The PF rat model was established using the bleomycin induction method (17). Rats were randomly allocated into four groups (*n* = 12) including a control (CON) group, model (MOD) group, positive drug control group (pirfenidone, 0.163 g/kg), based on the recommended dosage in the instruction manual and adjusted for body surface area (18), and drug delivery group. Following intraperitoneal anesthesia with 10% pentobarbital sodium (3.8 mL/kg), rats in each group were positioned supine on the experimental table with their heads and limbs immobilized. In both the model and treatment groups, a blunt needle was inserted through the oral cavity, passing between the tracheal cartilage rings, to administer 0.2 mL of bleomycin solution (approximately 5 mg/kg BLM) into the trachea. Subsequently, 0.2 mL of air was injected into the trachea multiple times, and the rats were promptly positioned upright and rotated to ensure uniform distribution of the drug in the lungs. In contrast, the control group received an equivalent volume of physiological saline through the trachea in identical circumstances. Following the animals’ natural recovery of consciousness, they were confined in cages for standard care procedures. Commencing on the initial day post-modeling, both the CON and MOD groups were orally administered an equivalent amount of physiological saline. The treatment group received SRL at the recommended dosage converted to rat proportions, once per day for approximately 4 weeks, at a dosage of 10 mL/kg. The administration of SRL continued for the specified 4-week period, during which the rats were monitored for overall health and mortality rates.

### 2.4 Sample collection

Following 4 weeks of uninterrupted administration, rats were euthanized via intraperitoneal injection of a 3% pentobarbital sodium solution (30 mg/kg) for abdominal anesthesia. Post-euthanasia, the characteristics of both lungs, such as color, texture, shape, and volume, were examined. Subsequently, 5 mL of blood was drawn from the abdominal aorta, with the serum being separated and stored at −80°C for metabolomics analysis. Take an appropriate amount of lung tissue, fix it with a 4% paraformaldehyde solution, freeze the remaining tissue in liquid nitrogen, and then store at a temperature of −80°C.

### 2.5 Determination of inflammatory factors and fibrosis-related indexes in rat lung tissues

Lung tissue samples of 0.1 g were collected from each group of rats, ground in 9-fold saline, and subsequently analyzed by enzyme-linked immunosorbent assay (ELISA) utilizing an ELISA reader, following the guidelines outlined in the corresponding assay kits. Essential biochemical markers such as IL-1β and IL-6, as well as fibrosis-associated indicators including HA, LN, PC-III, Col-IV, among others, were meticulously quantified and assessed in the lung tissue.

### 2.6 Observation of morphological changes in rat lung tissue

Fixed rat lung tissue was taken, embedded in paraffin and pathologic sections were made. Hematoxylin and eosin (H&E) staining and Masson staining were performed, and morphological changes in lung tissue were examined by microscopic analysis, and the results were subsequently recorded for pathological evaluation. Scoring was determined based on the extent of lesion, with criteria as follows: 0 points denoting absence of damage in H&E staining; 1 point indicating less than 25% affected area with mild fibrotic thickening of alveolar or bronchial walls, 2 points representing 26–50% affected area with moderate thickening of lung walls and absence of significant structural impairment, 3 points were assigned to samples indicating 51–75% damaged area with worsened fibrosis, noticeable lung structural damage, forming fibrotic bands or small fibrotic nodules, and 4 points were assigned to samples indicating more than 75% damaged area with severe distortion of structure and extensive fibrosis (19).

### 2.7 Transcriptomic analysis

Total RNA was extracted from lung tissue samples of the control group, model group, positive drug group, and treatment group using the Trizol reagent kit. Assessment of RNA sample degradation and contamination was conducted using 1% agarose gel electrophoresis and the concentration and purity of RNA (OD260/280 and OD260/230) were measured using a Nanodrop ultra-micro spectrophotometer. RNA integrity numbers (RIN) were determined using an Agilent Bioanalyzer 2,100 system, Santa Clara, CA, United States, and all samples met a threshold of ≥7.0. Additionally, purity was verified by spectrophotometric analysis, with the A260/A280 ratio consistently maintained between 1.8 and 2.0 for all samples. Specific oligonucleotide probes, such as polyT beads, were employed to selectively capture messenger RNA (mRNA) molecules by binding to the poly(A) tail at the 3′ end of mRNA. The enriched mRNA was subsequently converted into complementary DNA (cDNA) for library construction. The original sequencing data were obtained using the Illumina platform in FASTQ format, San Diego, CA, United States, and processed for mRNA identification and quantification using FASTP (0.19.7) software, Chen Runxin (Open Source, GitHub: OpenGene/fastp).Differential expression analysis was conducted utilizing the edgeR software, followed by Gene Ontology (GO) function enrichment analysis and Kyoto Encyclopedia of Genes and Genomes (KEGG) pathway enrichment analysis. The FASTP parameter settings: We processed the raw data (raw reads) using the FASTP (version 0.19.7) software. In this step, we obtained clean data (clean reads) by removing the following reads: (1) reads with 5′ adapters; (2) reads without 3′ adapters or insertion sequences; (3) reads with more than10% of N bases; (4) reads with more than 50% of bases with quality values lower than Qphred ≤ 20 reads; and (5) reads with multiple A/T/G/C. All downstream analyses were performed using clean, high-quality data. Clean reads from each sample were first mapped to the reference genome using the HISAT2 software, Johns Hopkins University, US. The read alignment results were transferred to the program String Tie for transcript assembly. The data format were used for the next analysis. We used transcript per million (TPM) for analysis: mRNA expression levels were estimated using the transcript per million (TPM) value as per the following criteria: normalization formula: Normalized expression = Mapped read countTotal reads × 1,000,000. For the samples with biological replicates, differential expression analysis of two conditions/groups was performed using the DESeq R package (1.8.3) (European Molecular Biology Laboratory (EMBL), Heidelberg, Germany). The *p*-values were adjusted using the Benjamini–Hochberg method. A corrected *p*-value of 0.05 was set as the threshold for significantly differential expression by default. A *p*-value < 0.05 and |log2 (foldchange)| (|log2FC|) > 1 was set as the threshold for significantly differential expression by default. Volcano plots and correlation heatmaps were generated using the Lianchuan BioCloud Platform^1^, Lianchuan Bio, China to visually represent the regulatory and clustering patterns of differentially expressed mRNAs (20). A significance threshold of *p*-value < 0.05 was employed for the differential expression analysis.

### 2.8 Metabolomics analysis based on UPLC-Q-TOF/MS

Serum samples were extracted, and data acquisition and analysis were carried out using ultra-performance liquid chromatography-quadrupole time-of-flight mass spectrometry (UPLC-Q-TOF/MS). UPLC-MS was performed on an ACQUITY UPLC HSS T3 C18 column (2.1 mm × 100 mm, 1.8 μm, Waters) (Waters Corporation, Milford, MA, United States) using two solvents [solvent A: 0.1% formic acid plus formic acid water (0.1%); solvent B: 0.1% formic acid plus acetonitrile] in the mobile phase: Isopropanol (1:1, v/v). The solvent gradient was as follows: (a) 0–3 min, 95% A and 5% B, changing to 80% A and 20% B; (b) 3–9 min, 80% A and 20% B, changing to 5% A and 95% B; (c) 9–13 min, 5% A and 95% B; (d) 13–13.1 min, 5% A and 95% B, changing to 95% A and 5% B; and (e) 13.1–16 min, 95% A and 5% B, used to equilibrate the system. The sample injection volume was 20 μL, the flow rate was 0.4 mL/min, and the column temperature was maintained at 40°C. Samples are stored at −4°C during analysis. Unsupervised principal component analysis (PCA) was performed utilizing Metabo Analyst software 6.0, McGill University, Canada, while supervised partial least squares discriminant analysis (PLS-DA) was executed with EZinfo 3.0 for Waters software, Umetrics, to reduce dimensionality and assess clustering separation within each group. The permutation test outcomes were evaluated using the Lianchuan BioCloud Platform (see text footnote 1) to ascertain the predictive capacity of the model and identify potential overfitting. Metabolites exhibiting statistically significant variances among the normal, model, and treatment groups of PF rats were identified through the application of variable importance in projection (VIP) > 2 and a significance level of *p* < 0.05. For metabolites, we used a combination of statistical significance (*p* < 0.05) and log2 foldchange (|log2FC| > 1) to determine differential metabolites. A Venn diagram analysis was subsequently conducted to ascertain the shared differential metabolites between the normal and model groups, as well as between the model and treatment groups. Additionally, a correlation heatmap analysis was performed to evaluate the clustering correlation of these identified differential metabolites. Subsequently, the Metabo Analyst software version 6.0 was utilized for the analysis of metabolic pathways in order to identify significant metabolic pathways associated with PF (−Logp > 2, Impact > 0.02). Key biomarkers impacted by treatment were selected based on the differential metabolites identified within these pathways.

### 2.9 Joint analysis of transcriptomics and metabolomics

The Cytoscape 3.7.0 plugin Metscape was employed for the construction and analysis of a network depicting the relationships between genes and metabolites. Gene and metabolite identities (IDs) were imported into Metscape to investigate the connections between changes in genes and metabolites, thereby elucidating the potential mechanisms of action of SRL in the treatment of PF. The KEGG database^2^ was utilized for the analysis of the biological processes associated with differentially expressed genes and metabolites, as well as the regulatory interactions between genes and metabolites.

### 2.10 Statistical analysis

Statistical analysis of the experimental data was conducted using Statistical Package for Social Sciences (SPSS) version 26.0 software (IBM, Armonk, NY, United States) with data presented as mean ± standard deviation (x̅ ± s). One-way analysis of variance (ANOVA) was used for comparisons among multiple groups, and pairwise comparisons within groups were conducted using the Wilcoxon rank sum test. *p* < 0.05 indicates that the difference is statistically significant. The R version is R 4.3.0, R Core Team (Open Source) and the following packages were used: “ggplot2,” “dplyr,” and 'biomaRt.”

## 3 Results

### 3.1 Changes in the content of inflammatory factors and fibrosis-related indexes in rat lung tissues

**Figure 1:**
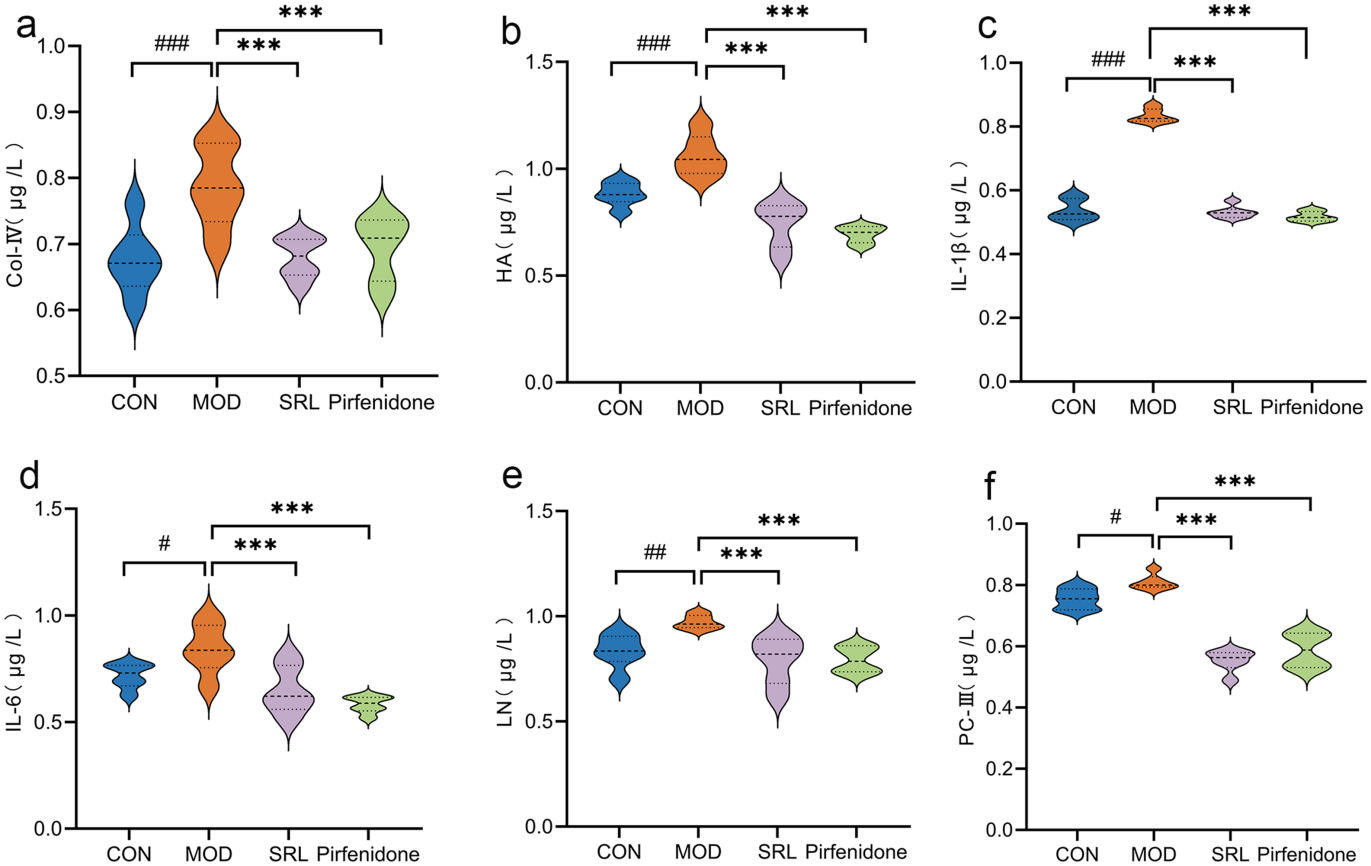
The changes in the content of inflammatory cytokines and fibrosis-related markers within the pulmonary tissue of rats. **(a)** COL-IV. **(b)** HA. **(c)** IL-1β. **(d)** IL-6. **(e)** LN. **(f)** PC-III. CON, control group; MOD, model group; SRL, Saorilao-4 decoction; Pirfenidone: pirfenidone positive drug group. Compared with the normal control group, ^####^*p* < 0.001; ^###^*p* < 0.01; ^#^*p* < 0.05 compared with the model group, ***p < 0.001; **p < 0.01; *p < 0.05.

Compared with the CON group, the levels of IL-6 and procollagen type III (PC-III) in the lung tissues of rats in the MOD group were significantly higher (*p* < 0.05). Additionally, laminin (LN), hyaluronic acid (HA), collagen IV (Col IV), and IL-1β levels exhibited substantial increases (*p* < 0.01 or *p* < 0.001). In contrast, compared with the MOD group, the levels of IL-6, LN, Col IV, HA, PC-III, and IL-1β in the lung tissues of rats in the SRL group and the pirfenidone group were significantly reduced (*p* < 0.001), close to the levels of the normal group (Figure 1). These findings suggest that the pulmonary fibrosis (PF) model was successfully established in the MOD group, and SRL demonstrated anti-inflammatory properties and effectively controlled and mitigated PF.

### 3.2 Morphologic changes in rat lung tissue

**Figure 2:**
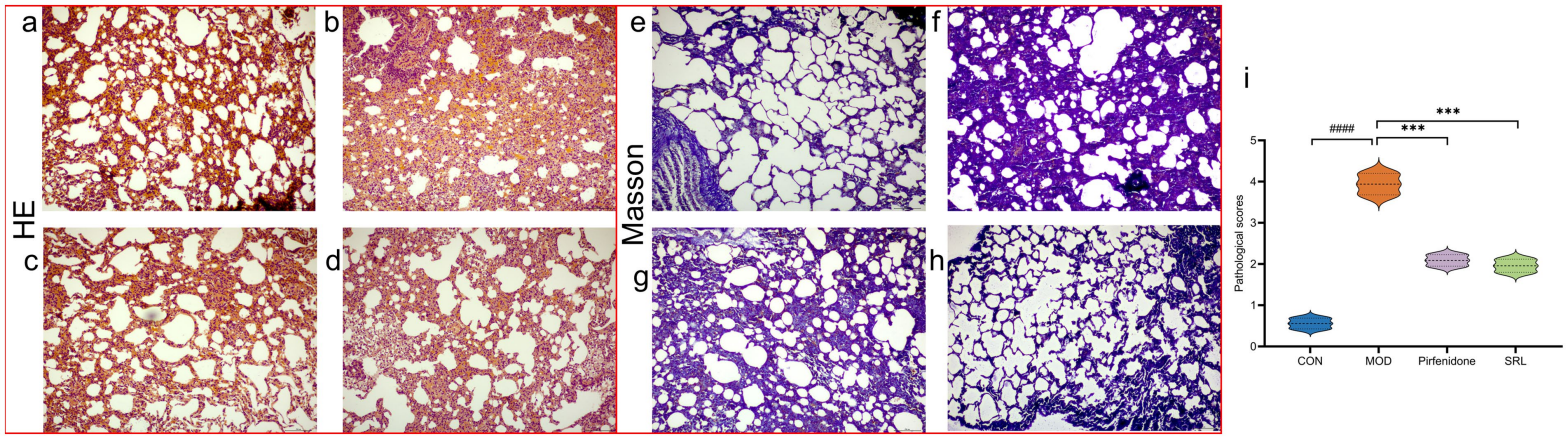
**(a–d)** Micrographs of H&E-stained lung tissue. **(e–h)** Masson-stained lung tissue micrographs. **(i)** Lung histopathological scoring diagrams CON: control group; MOD: model group; SRL: Saorilao-4 decoction; Pirfenidone: pirfenidone positive drug group. Compared with normal control group, ###*p* < 0.001; compared with model group, ***p < 0.001.

The H&E-stained lung tissue sections of rats in CON (Figure 2a) revealed well-preserved alveolar structures, no fatty degeneration, and inconspicuous inflammatory responses. In contrast, the H&E-stained lung tissue sections of rats in MOD (Figure 2b) displayed pronounced alveolar inflammation, thickened septa, and a noticeable infiltration of inflammatory cells around the alveolar interstitium, with a significant increase in pathological scores (*p* < 0.001) (Figure 2i). However, the positive drug control group (Figure 2c) and the SRL treatment group (Figure 2d) showed a significant reduction in inflammatory cell infiltration in the lung interstitium, improved alveolar structure, and a significant decrease in pathological scores (*p* < 0.001). The Masson’s trichrome staining results indicated that the lung tissue structure of the control group mice was normal, with no visible blue collagen fiber deposition (Figure 2e). Compared to the CON group, the MOD group’s mice exhibited extensive blue collagen fiber deposition in the lung tissue and septa (Figure 2f). The positive drug CON (Figure 2g) and the SRL treatment group showed a relative reduction in blue collagen fiber deposition, indicating an improvement in fibrosis symptoms (Figure 2h). These results suggest that the rat PF model was successfully established in this study, and treatment with SRL resulted in the repair of alveolar structures, alleviation of inflammatory responses, reduction of collagen deposition, and a significant improvement in PF.

### 3.3 Transcriptomics results of SRL-treated PF rats

**Figure 3:**
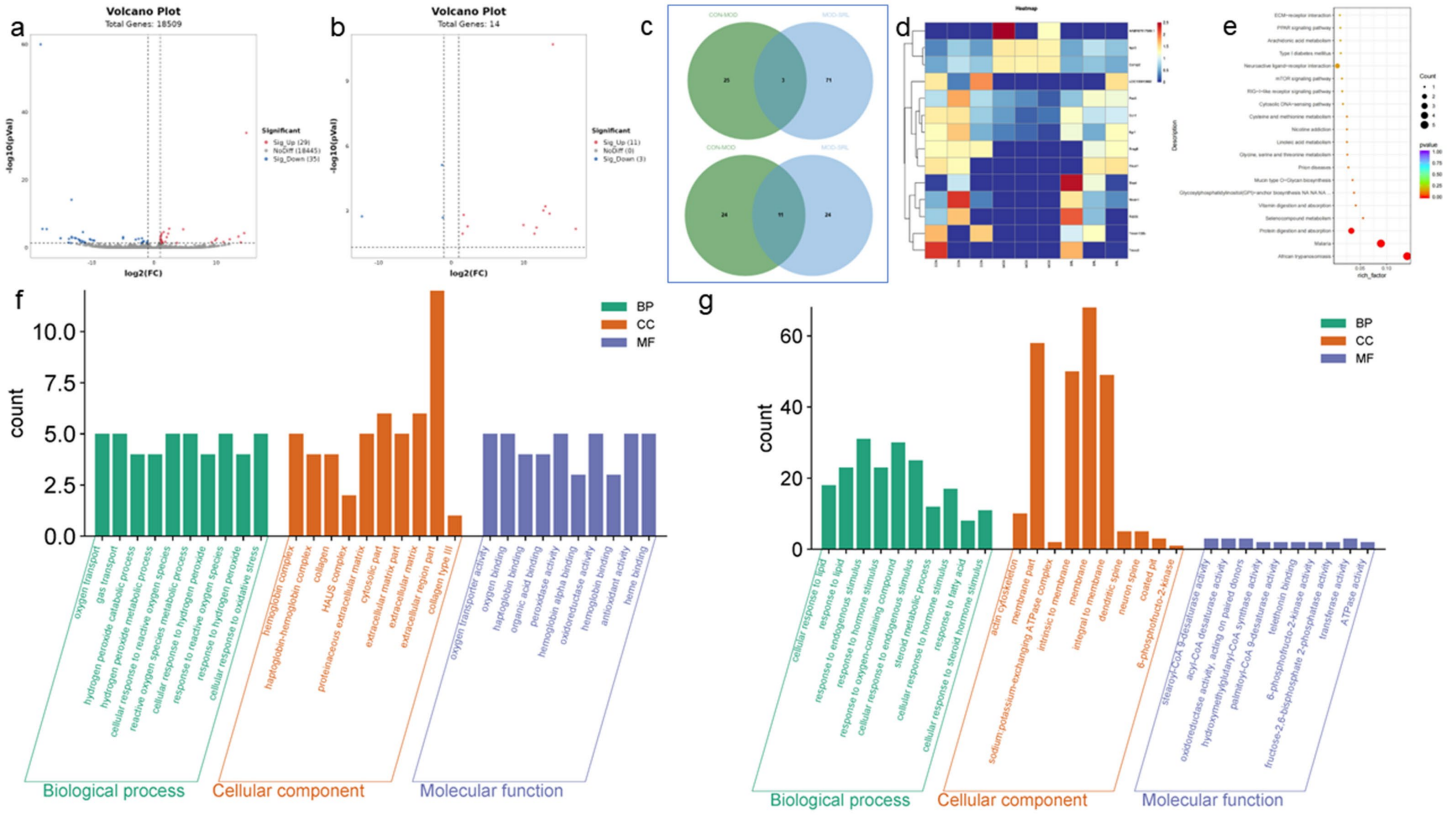
Results of transcriptomic analysis of SRL-administered PF rats. **(a)** Volcano plots of DEGs in CON and MOD groups. **(b)** Volcano plots of DEGs after SRL administration. **(c)** CON vs. MOD upregulated genes vs. MOD vs. SRL downregulated genes Venn diagram plot; CON vs. MOD downregulated genes vs. MOD vs. SRL upregulated genes Venn diagram plot. **(d)** Heatmap visualization of the intensity of 14 DEGs with restored expression. **(e)** KEGG pathway analysis based on transcriptomics. **(f)** CON-MOD GO enrichment analysis of DEGs’ biological processes. **(g)** MOD-SRL GO enrichment analysis of DEGs’ biological processes. CON, control group; MOD, model group; SRL, Saorilao-4 decoction.

Differential expression genes (DEGs) were identified and analyzed in CON, MOD, and SRL groups. The volcano plot (Figure 3a) revealed 64 DEGs that exhibited significant expression differences between the control and model groups, with 29 upregulated and 35 downregulated genes (Table 1). Subsequent to SRL administration, the expression levels of 14 DEGs were normalized (Figure 3b), consisting of 11 upregulated and 3 downregulated genes (Figures 3c,d). These findings suggest that SRL treatment effectively restored the aberrant expression of certain genes to baseline levels. To provide a more comprehensive understanding of the potential mechanisms driving the alterations in differentially expressed genes (DEGs), all candidate biomarkers underwent KEGG enrichment and functional analysis. The primary enriched pathways observed at the transcriptional level in the CON and MOD groups (Figure 3e) encompassed protein digestion and absorption, peroxisome proliferator-activated receptors (PPAR) signaling pathway, glycine, serine, and threonine metabolism, as well as arachidonic acid metabolism. Gene ontology (GO) analyses were subsequently performed to further investigate the functional roles of the genes in the DEGs between the CON and MOD groups (Figure 3f) and between the MOD and SRL groups (Figure 3g). Go Enrichment analyses showed enrichment in terms such as negative regulation of transcription from RNA polymerase, cellular response to lipid, and response to fatty acid.

**Table 1:** Sixty-four dysregulated genes.

Gene	log2foldchange	padj	C/M	M/S
Mboat7	−18.2622	1.3229E-34	↓	↑
Alox15	−13.2853	2.7011E-06	↓	↑
LOC100911572	−17.9720	3.5935E-06	↓	
Retnlg	−17.2920	4.1100E-06	↓	
Npr3	−11.4464	2.8422E-05	↓	
Serpina3n	−13.2722	7.8929E-04	↓	
Sirpb3	−4.98121	8.5108E-04	↓	
Haus1	−13.2336	1.4084E-03	↓	
Ucp1	−12.7522	1.8044E-03	↓	↑
Hbb-b1	−15.0514	1.8044E-03	↓	
Ccn1	−12.5727	1.9246E-03	↓	
AABR07065837.1	−1.95077	2.1210E-03	↓	↑
Upk3a	−4.98258	2.4072E-03	↓	
LOC100911881	−2.48500	2.7837E-03	↓	↑
Gpr156	−13.7158	3.3489E-03	↓	
Cemip2	−10.3400	3.3964E-03	↓	
XLOC_114505	−12.1360	4.5021E-03	↓	↑
Nkain1	−2.95975	5.1706E-03	↓	
Dnaja1	−10.1900	5.5876E-03	↓	↑
AABR07067896.1	−11.9152	7.5869E-03	↓	
AABR07000740.1	−9.8156	8.7209E-03	↓	
LOC103692170	−9.6215	1.1896E-02	↓	
Sirpd	−11.5414	1.2786E-02	↓	
Pax5	−11.9464	1.2931E-02	↓	↑
Ptpru	−1.7209	1.4570E-02	↓	↑
Irf7	−1.0765	1.4570E-02	↓	
LOC108348074	−1.6487	1.6277E-02	↓	
Rpl35al1	−1.7984	2.2565E-02	↓	
Clic2	−11.1747	2.4507E-02	↓	↑
LOC100910143	−11.5550	2.8185E-02	↓	
Celf6	−13.7794	4.0355E-02	↓	↑
Cpe	−11.1176	4.3230E-02	↓	
AABR07051689.1	−1.14009	4.3230E-02	↓	
Rit2	−2.0563	4.6774E-02	↓	
Hba-a2	−13.3893	4.8547E-02	↓	
RragB	14.8705	7.6319E-15	↑	
LOC108348078	2.4424	3.2845E-06	↑	
LOC691918	4.6996	2.8114E-05	↑	
Cpne1	1.5592	5.5822E-05	↑	↓
Ano2	14.5252	7.5979E-05	↑	
Adamts9	2.2731	7.9968E-05	↑	
LOC103694855	1.21111	2.4266E-04	↑	
AABR07065656.5	1.1453	5.7661E-04	↑	
LOC100134871	13.5905	6.0946E-04	↑	
Il1rapl1	1.0641	6.7616E-04	↑	
Tmprss11a	2.0485	1.3601E-03	↑	
Nhp2	1.0578	1.9246E-03	↑	
Egr1	10.0871	2.6902E-03	↑	
AABR07017599.1	1.0495	3.7221E-03	↑	↓
Tmem132b	12.1878	3.7221E-03	↑	↓
Scn3b	1.1796	6.4694E-03	↑	
Gabra3	1.0530	7.5103E-03	↑	
Slc19a3	9.8566	7.7327E-03	↑	
Loxl1	1.2731	1.7853E-02	↑	
LOC103694857	1.1551	1.8770E-02	↑	
Ephb1	1.2230	2.0075E-02	↑	
AABR07051551.2	1.8136	2.2346E-02	↑	
Rab3c	11.1851	2.3097E-02	↑	
Col15a1	1.3347	2.5200E-02	↑	
Tprg1	1.3034	2.6206E-02	↑	
LOC103691744	9.2761	2.7916E-02	↑	
Tmco3	13.9733	3.4254E-02	↑	
AABR07034730.3	3.6730	4.5192E-02	↑	

C, CON, normal group; M, MOD, model group; S, SRL, Saorilao-4 decoction administration group.

### 3.4 Results of metabolomics analysis

**Figure 4:**
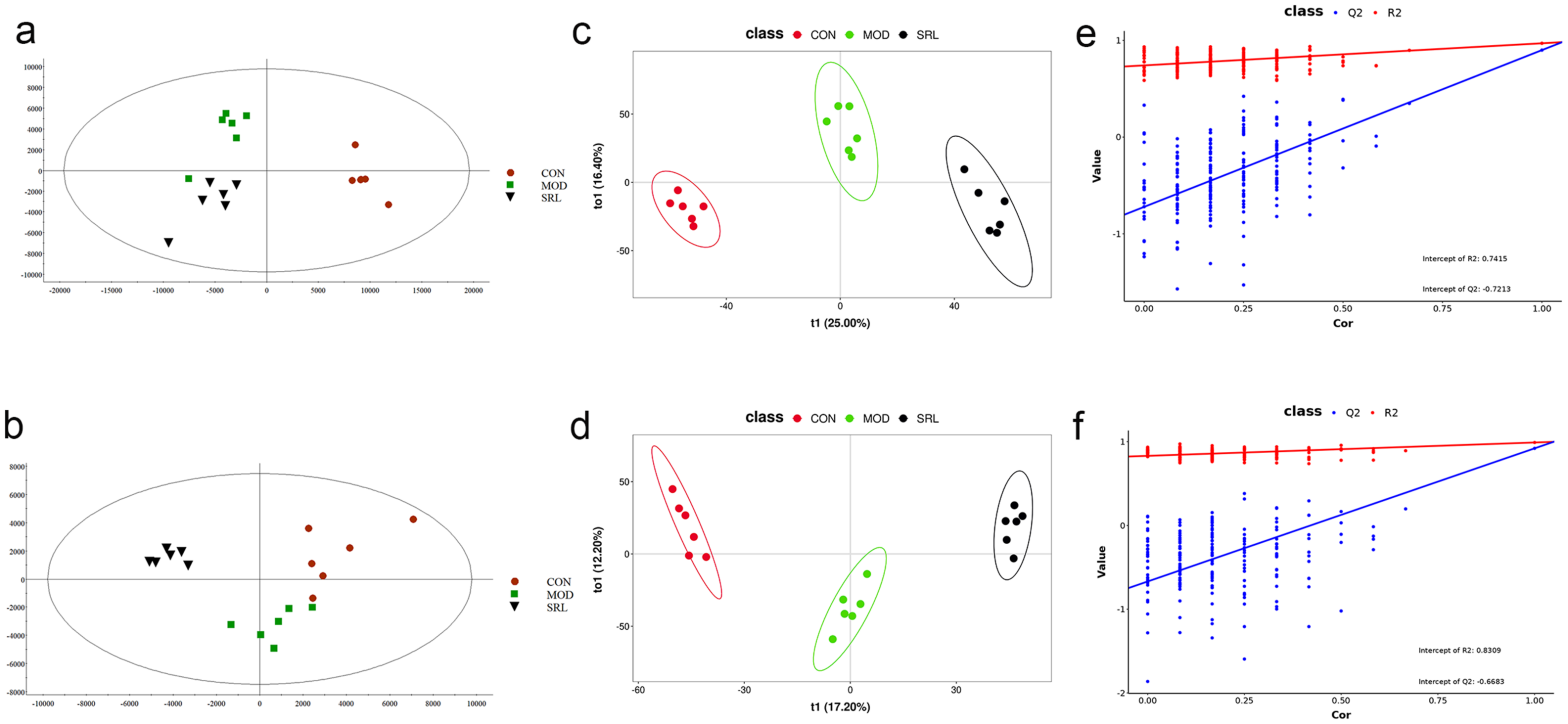
Analysis of metabolomic results in SRL-administered PF rats. **(a,b)** Principal component analysis (PCA scatter plots) of serum metabolites based on CON and MOD groups in positive and negative ion modes. **(c,d)** PLS-DA scatter plots are plotted in positive and negative ion modes, respectively. **(e,f)** PLS-DA model score plots validated by class alignment test in positive and negative ion modes, respectively. CON, control group; MOD, model group; SRL, Saorilao-4 decoction.

**Figure 5:**
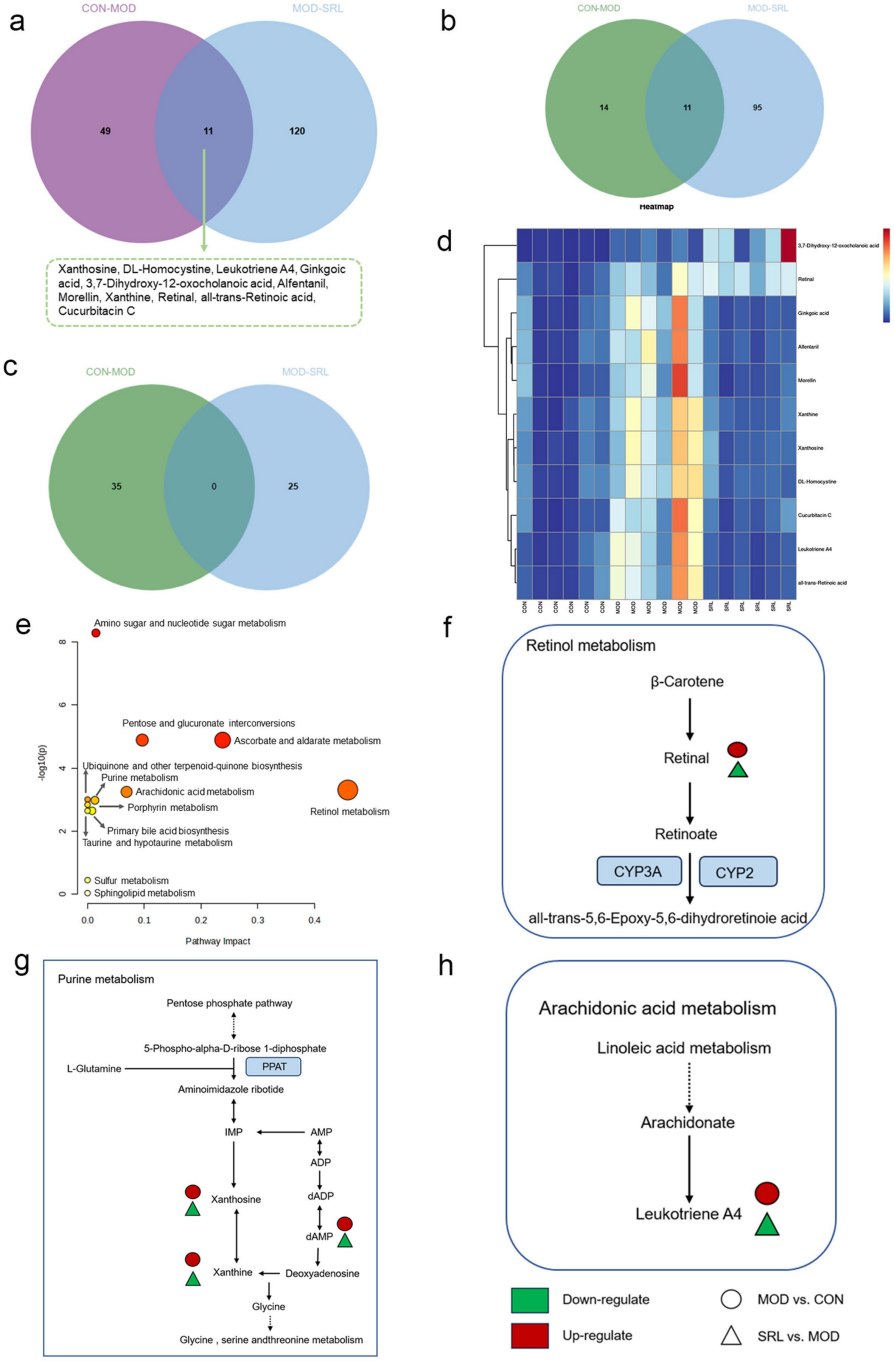
**(a)** Venn diagram of differential metabolites between CON and MOD groups, and MOD and SRL groups. **(b)** CON vs. MOD upregulated genes vs. MOD vs. SRL downregulated genes Venn diagram plot. **(c)** CON vs. MOD downregulated genes vs. MOD vs. SRL upregulated genes Venn diagram plot. **(d)** Heatmap visualization of the levels of 11 metabolic biomarkers in different groups. Color saturation indicates metabolite expression values, with blue indicating the lowest expression and red the highest. **(e)** Overview of pathway analysis at the metabolomic level of PF. **(f–h)** Schematic overview of metabolic pathway changes in PF model and SRL treatment. CON, control group; MOD, model group; SRL, Saorilao-4 decoction.

To examine the metabolic traits of the PF model and SRL-treated rats, UPLC-QTOF-MS was utilized to analyze the metabolic profiles of serum samples from various rat groups in both positive and negative electrospray ionization (ESI) modes. Multivariate analysis was performed using EZinfo 3.0 for Waters software on the data extracted from the QI software (Waters Corporation, Milford, MA, United States). Unsupervised PCA and supervised PLS-DA were employed to distinguish the classifications of all metabolic samples. The PCA score plots (Figures 4a,b) illustrated notable differentiation of metabolic states. Furthermore, a high level of intra-group clustering was observed, suggesting strong reproducibility and consistency in the experiments, as well as the stability and reliability of the models and treatment modalities within each group. Similarly, the PLS-DA score plots (Figures 4c,d) demonstrated significant differentiation among the CON, MOD, and SRL groups. The PLS-DA cross-validation results (Figures 4e,f) showed R2 and Q2 values of 0.74 and −0.72, respectively, in the positive ion mode, and 0.83 and −0.66, respectively, in the negative ion mode. Q2 represents the predictive performance of the model as determined by cross-validation, while R2 signifies the model’s goodness of fit. High values of R2 and Q2, approaching 1, indicate a well-constructed model. Based on multivariate analysis using MetaboAnalyst 6.0, 60 potential biomarkers were identified between the MOD and CON groups after filtering with the criteria of VIP > 2 and *p* < 0.05 (Table 2). We found that, compared to the CON group, 25 metabolites were upregulated and 35 metabolites were downregulated in the MOD group. Furthermore, 131 differential metabolites were identified between the MOD group and SRL, with 11 of these metabolites also exhibiting differences between the MOD and CON groups (Figure 5a). The levels of the 11 differential metabolites displayed notable variations among the groups, indicating regulatory influences at the metabolic level (Figures 5b–d). Further elucidation of the potential mechanisms underlying these metabolite changes involved subjecting all potential biomarkers to enrichment and functional analysis using metabolomics pathway analysis (MetPA), which highlighted significant alterations in major metabolic pathways between the CON group and MOD group at the metabolomic level (Figure 5e). The aforementioned results indicate that the progression of PF disrupts potential biomarkers such as UDP-glucuronate, dAMP, Retinal, Leukotriene A4 and Taurocholate, triggering metabolic pathway disorders in amino sugar and nucleotide sugar metabolism, ascorbic acid and aldehyde acid metabolism, pentose and glucuronic acid interconversion, retinol metabolism, arachidonic acid metabolism, purine metabolism, primary bile acid biosynthesis, and sphingolipid metabolism. SRL administration exhibits a strong regulatory effect on metabolites such as Xanthosine, dAMP, Retinal, and Leukotriene A4, which are primarily involved in arachidonic acid metabolism, purine metabolism, and retinol metabolism, and affect energy metabolism and transport, thereby influencing lipid regulatory functions (Figures 5f–h).

**Table 2:** The regulatory relationships of 60 metabolites in CON and MOD groups and their variations in the SRL group.

NO	Metabolites	Ratio	VIP	M/Z	Ion mode	MS1 keggID	Regulated (M/C)	Regulated (S/M)
1	Rhamnose	13.50372	2.353296	163.0615	−	C00507	↑	
2	2(3H)-Benzothiazolethione	0.027958	2.570221	165.9796	−	C14437	↓	
3	O-Phosphohomoserine	0.06345	2.835044	198.0159	−	C01102	↓	
4	Xanthosine	5.837372	2.613518	283.0689	−	C01762	↑	↓
5	DL-Homocystine	4.897652	2.56177	284.0721	−	C01817	↑	↓
6	Leukotriene A4	6.38739	2.344073	317.2125	−	C00909	↑	↓
7	Brompheniramine	5.247961	2.066524	317.0648	−	C06857	↑	
8	Dolasetron	14.02756	2.535233	323.1402	−	C07866	↑	
9	Deoxyadenosine monophosphate	13.07308	2.297281	330.0618	−	C00360	↑	
10	Ginkgoic acid	5.228546	2.275503	345.2438	−	C10794	↑	↓
11	3,7-Dihydroxy-12-oxocholanoic acid	7.79384	2.14935	405.2648	−	C01292	↑	↓
12	Alfentanil	4.709391	2.376845	415.2464	−	C08005	↑	↓
13	Estradiol-17alpha 3-D-glucuronoside	10.57509	2.29912	483.1762	−	C04300	↑	
14	Morellin	5.75644	2.534897	560.2638	−	C10085	↑	↓
15	Urobilinogen	7.628563	2.81009	606.3341	−	C05791	↑	
16	Fumonisin B1	0.085677	2.641306	720.3773	−	C19241	↓	
17	Amphotericin B	0.086825	2.620882	939.5058	−	C06573	↓	
18	Xanthine	5.252805	2.486882	153.0409	+	C00385	↑	↓
19	(S)-Homostachydrine	0.205786	2.13019	158.1173	+	C08283	↓	
20	Retinal	4.989705	2.248402	285.2208	+	C00376	↑	↓
21	dAMP	6.512208	2.38212	301.2162	+	C00777	↑	↓
22	Quinine	0.22598	2.038241	325.1936	+	C06526	↓	
23	Phytanic acid	0.120212	2.448753	351.2636	+	C01607	↓	
24	Betazole	10.85833	2.608791	359.1489	+	C05366	↑	
25	Disopyramide	0.183429	2.206751	362.2224	+	C06965	↓	
26	Clemastine	11.19952	2.378317	382.1341	+	C06913	↑	
27	Mammeisin	5.714011	2.05327	407.1886	+	C09275	↑	
28	(±)-Naled	0.225565	2.044597	416.7482	+	C18749	↓	
29	Lovastatin acid	0.226959	2.053382	423.2744	+	C21130	↓	
30	Vitamin K1 2,3-epoxide	0.097315	2.272284	467.3475	+	C05849	↓	
31	Loperamide	0.220275	2.078937	477.2305	+	C07080	↓	
32	Galactosylsphingosine	0.23041	2.008711	500.2955	+	C01747	↓	
33	Deoxyadenosine triphosphate	0.177156	2.123148	513.9879	+	C00131	↓	
34	2’-Deoxyinosine triphosphate	0.195154	2.024276	514.9709	+	C01345	↓	
35	Cucurbitacin I	0.185398	2.265518	515.3041	+	C08800	↓	
36	Taurocholic acid	0.131195	2.391303	516.3013	+	C05122	↓	
37	Phosphoadenosine phosphosulfate	0.191753	2.02459	529.9802	+	C00053	↓	
38	Fusidic Acid	5.607628	2.151472	534.3739	+	C06694	↑	
39	UDP-4-dehydro-6-deoxy-D-glucose	39.75498	3.193859	549.0497	+	C04089	↑	
40	Cucurbitacin B	0.167447	2.278903	559.331	+	C08794	↓	
41	Cucurbitacin C	7.393605	2.847709	561.3374	+	C08795	↑	↓
42	Daunorubicin	99.5055	3.648724	566.144	+	C01907	↑	
43	Ceftiofur	0.149456	2.394545	567.9966	+	C13143	↓	
44	Cotinine glucuronide	0.044092	2.970441	575.01	+	C00190	↓	
45	Hyperforin	0.074896	2.722633	575.3465	+	C07608	↓	
46	all-trans-Hexaprenyl diphosphate	0.199306	2.048638	587.3287	+	C01230	↓	
47	Uridine diphosphate glucuronic acid	0.086516	2.542274	603.0232	+	C00167	↓	
48	1-Phenylheptane	0.106715	2.499087	604.3538	+	C11954	↓	
49	Uridine diphosphate-N-acetylglucosamine	11.75029	2.300309	608.0852	+	C00043	↑	
50	Phosphoribosylformimino-AICAR-P	0.063208	2.777815	616.0469	+	C04896	↓	
51	Astaxanthin	0.115744	2.417381	619.3718	+	C08580	↓	
52	Remikiren	0.162004	2.271763	631.3556	+	C07465	↓	
53	1,2,6-Trigalloyl-beta-D-glucopyranose	23.9315	2.888278	637.1017	+	C04360	↑	
54	Calenduloside E	0.090185	2.622698	655.3818	+	C08964	↓	
55	D-altro-D-manno-Heptose	0.196481	2.009331	658.0564	+	C06397	↓	
56	Cyanidin 3-(3″,6″-dimalonylglucoside)	0.06701	2.738531	660.0726	+	C16289	↓	
57	Amiodarone	0.057964	2.877233	663.0626	+	C06823	↓	
58	Digitoxin	0.059092	2.839339	765.4462	+	C06955	↓	
59	Gymnemic acid I	0.095909	2.540749	824.4852	+	C08947	↓	
60	Troleandomycin	0.077636	2.68799	831.4866	+	C12753	↓	

VIP, variable importance in projection; VIP values from PLS-DA model; M/Z, mass-to-charge ratio; C, CON, normal group; M, MOD, model group; S, SRL, Saorilao-4 decoction administration group.

### 3.5 Integrated analysis of transcriptomic and metabolomics

**Figure 6:**
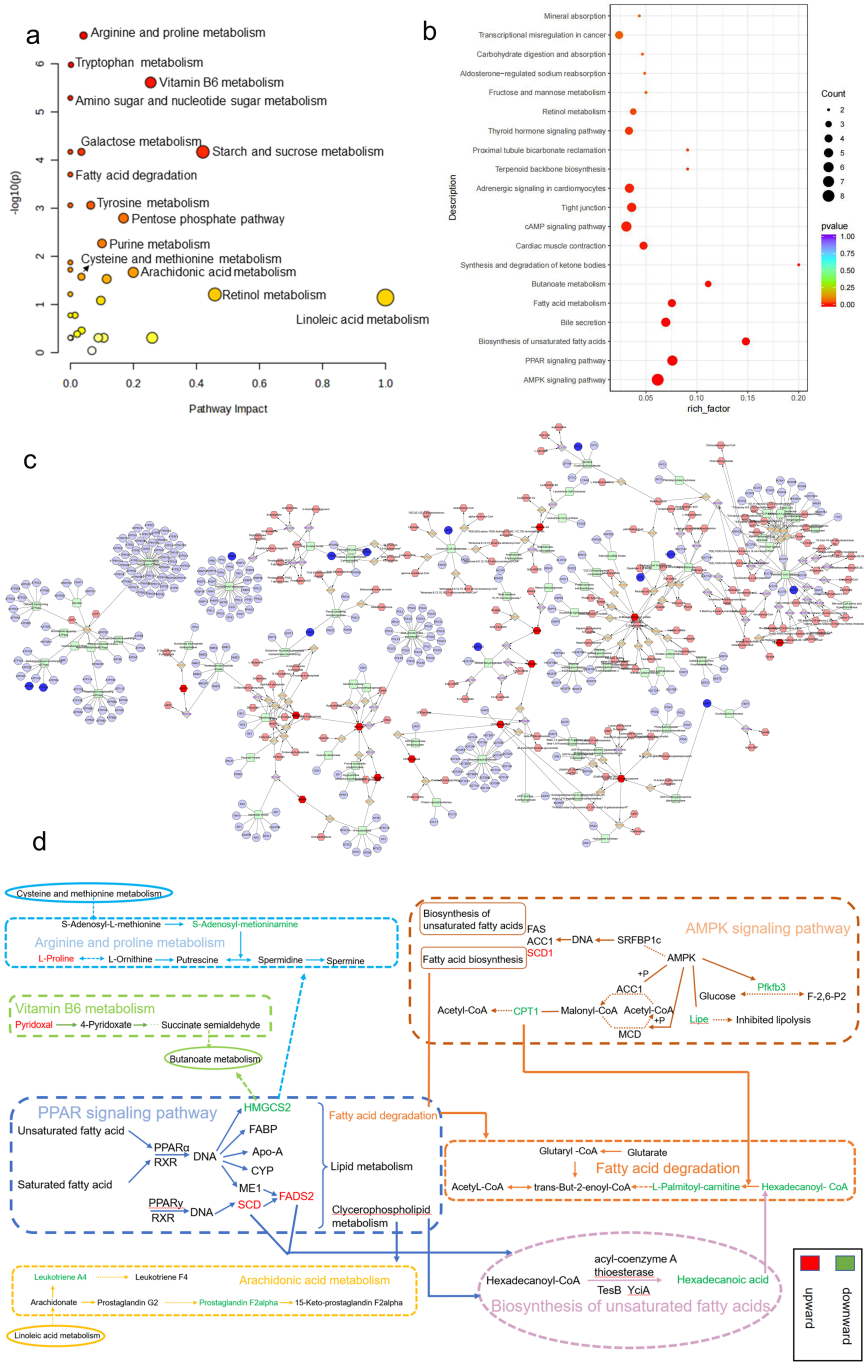
Results of combined transcriptomics and metabolomics analysis in SRL-administered PF rats. **(a)** Pathways analyzed for KEGG enrichment at the metabolic level in SRL-administered PF rats. **(b)** Pathways analyzed for KEGG enrichment at the gene level in SRL-administered PF rats. **(c)** Metscape analysis results mainly related to lipid metabolism, protein metabolism, and glucose metabolism. **(d)** Combined pathway analysis based on transcriptomics and metabolomics. Colors indicate the trend of regulation of differential metabolite and differentially expressed gene content; red indicates upregulation and green indicates downregulation. CON: control group; MOD: model group; SRL: Saorilao-4 decoction.

Using the KEGG comprehensive database, a pathway-associated analysis was conducted to examine gene and metabolite functional enrichment. Following treatment with SRL, the modulation of biomarkers such as S-adenosylmethioninamine (SAM), 16-hydroxyhexadecanoic acid and proline helped alleviate disruptions in various pathways, including arginine and proline metabolism, tryptophan metabolism, vitamin B6 metabolism, amino sugar and nucleotide sugar metabolism, fatty acid degradation, arachidonic acid metabolism, cysteine and methionine metabolism, and primary bile acid biosynthesis (Figure 6a), ultimately leading to a certain degree of symptom relief. Further investigation into gene regulation following SRL administration was carried out using KEGG pathway enrichment analysis (Figure 6b). The MOD group exhibited significant upregulation of genes such as *Scd*, *Scd2*, and *Fads2*, as well as downregulation of *Cpt1a*, *Lipe*, *Pfkfb3*, and *Hmgcs2* after SRL ingestion. These genes are primarily linked to the adenosine monophosphate-activated protein kinase (AMPK) signaling pathway, PPAR signaling pathway, unsaturated fatty acid biosynthesis, and fatty acid metabolism. To assess the specific metabolic pathways impacted by SRL in the lungs of PF rats, differential metabolites and genes were integrated into Cytoscape for Metscape analysis for further examination. The gene-metabolite correlation network predominantly involved various metabolic pathways, such as lipid metabolism, amino acid metabolism, and carbohydrate metabolism. These pathways included arachidonic acid metabolism, fructose and mannose metabolism, galactose metabolism, glycine, serine, threonine, and alanine metabolism, glycolysis and gluconeogenesis, linoleic acid metabolism, lysine metabolism, monounsaturated fatty acid *β*-oxidation, omega-3 fatty acid metabolism, omega-6 fatty acid metabolism, pentose phosphate pathway, purine metabolism, saturated fatty acid β-oxidation, tyrosine metabolism, urea cycle, and metabolism of arginine, proline, glutamate, aspartate, and asparagine, as well as vitamin metabolism (Figure 6c). The findings suggest a strong correlation between lipid metabolism and metabolic pathways in the context of SRL therapy for PF. Furthermore, analysis using the KEGG comprehensive database revealed regulatory connections between core genes *FADS2*, *CPT1*, and *SCD* and differential metabolites such as hexadecanoic acid and proline during PF treatment. Our research findings indicate that SRL has the potential to modulate the expression levels of key genes, including *FADS2*, *CPT1*, and *SCD*, by activating the AMPK and PPAR signaling pathways, thereby influencing the biosynthesis of unsaturated fatty acids and fatty acid metabolism pathways (Figure 6d).

## 4 Discussion

**Figure 7:**
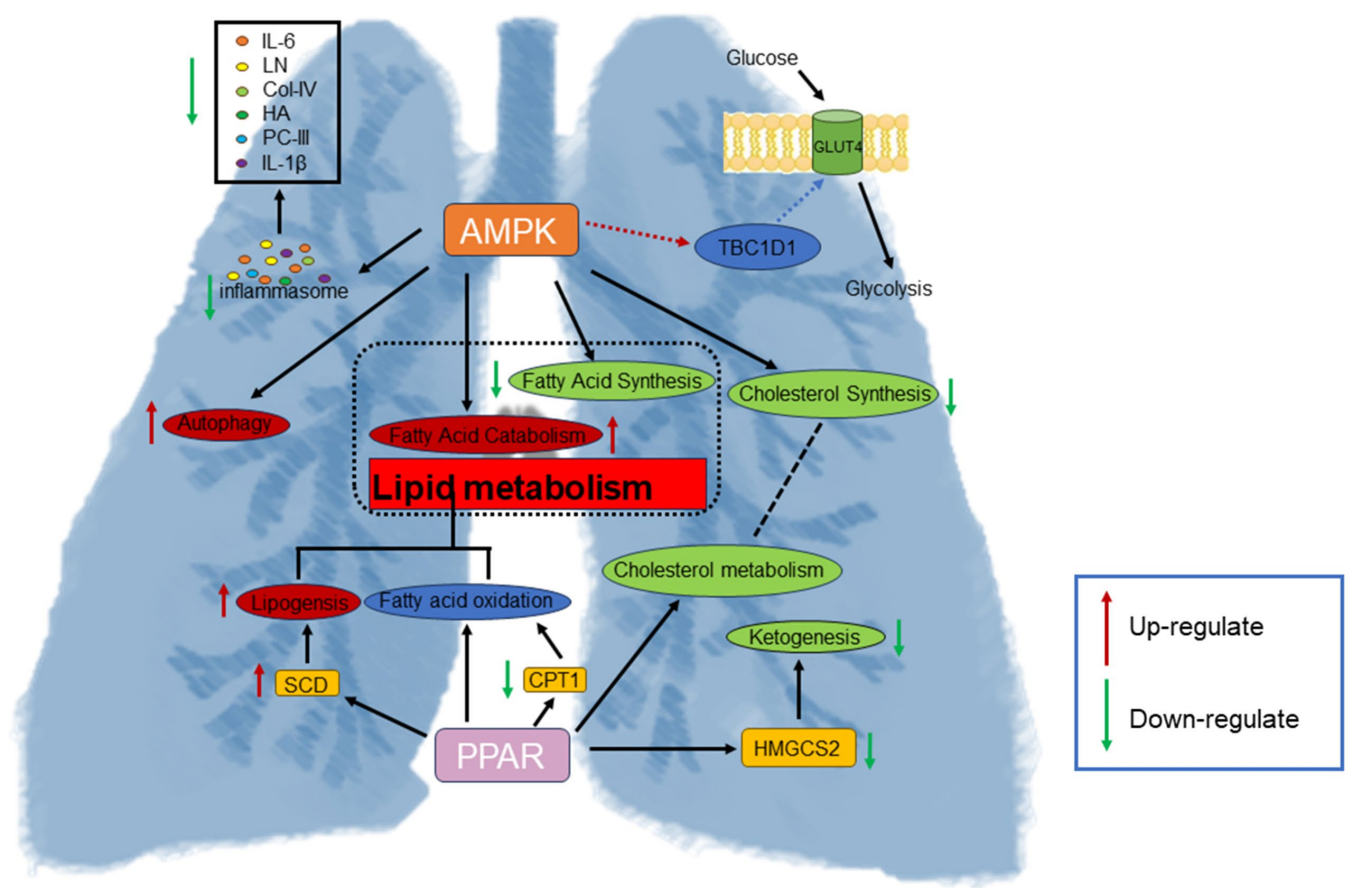
SRL activates the PPAR signaling pathway and AMPK signaling pathway to regulate fatty acid metabolism, increase the regulation of energy homeostasis, reduce inflammatory stress, and alleviate the development of PF.

Omics technologies are employed to systematically and comprehensively investigate the overall effects of traditional Mongolian medicine (TMM) and explore the dynamic characteristics of pharmacological systems in therapy (21–23). In this study, TMM was utilized as a treatment strategy aimed at providing a comprehensive and systemic understanding of the complex pathophysiological processes of PF. The study selected 8-week-old rats, typically at a stable physiological development stage. During this period, the organs and systems of rats have matured, and their physiological functions are relatively stable (24). The metabolic level of 8-week-old rats is stable, which can better reflect the changes in metabolites under specific experimental conditions (25), whose expression changes are closely related to the development of respiratory diseases. In 8-week-old rats, specific gene expression changes may relate to respiratory disease development (26). Furthermore, we employed a multi-omics approach combining transcriptomics and metabolomics to explore the therapeutic mechanisms of SRL in PF, with a focus on the regulation of energy homeostasis based on amino acid and fatty acid metabolism, along with the associated differentially expressed genes (DEGs) and metabolites. This approach helps understand TMM’s mechanisms in PF treatment, offering a scientific basis for future research and therapies (Figure 7).

IL-6 and IL-1β play key roles in immune regulation and inflammatory responses. Elevated levels of these cytokines can promote fibroblast proliferation and collagen synthesis, contributing to the development of fibrosis (27). PC-III, Col-IV, and LN are essential structural components of the basement membrane, crucial for cell adhesion and signal transduction (28). Upregulation of these components in PF can disrupt normal basement membrane repair processes, worsening fibrotic progression (29). Hyaluronic acid (HA), a common polysaccharide in the extracellular matrix, may influence tissue repair and fibrosis accumulation during inflammation and fibrosis (30). Experimental findings show a significant rise in serum markers in the MOD group, reduced to levels similar to the positive drug and CON groups after SRL administration. Histological analyses indicated considerable lung damage in the MOD group versus healthy tissue. However, after SRL treatment, this damage was notably attenuated, restoring lung tissue morphology to a state resembling that of the CON group and positive drug group. These collective experimental results confirm the successful establishment of a bleomycin-induced rat model of PF and highlight the beneficial effects of SRL in ameliorating and treating PF.

Moreover, 60 potential metabolic biomarkers linked to PF were screened, revealing that after SRL treatment, 11 biomarkers returned to normal levels. Decreased. Notably, in normal rats, 64 genes related to PF showed significant expression levels. A detailed analysis using MetaboAnalyst 6.0 revealed insights into PF’s pathophysiology and SRL’s mechanism. The study results suggest that SRL may exert its therapeutic effects on PF by modulating amino acid and fatty acid metabolism. Major metabolic pathways implicated include arginine and proline metabolism, vitamin B6 metabolism, fatty acid degradation, arachidonic acid metabolism, and primary bile acid biosynthesis. The PPAR and AMPK signaling pathways are key in SRL’s therapeutic effects on PF. These findings offer valuable clues and references for a deeper comprehension of PF pathogenesis and the therapeutic effects of SRL.

At the metabolite level, SRL impacts vitamin B6 metabolism. Previous studies have shown that vitamin B6 can regulate tumor cells’ responses to various stress conditions (31). Decreased circulating levels of vitamin B6 are often associated with hyperhomocysteinemia, a known risk factor for PF (32). The level of pyridoxal metabolism was significantly increased in the SRL-administered group, thereby increasing the circulating levels of vitamin B6. In arginine and proline metabolism, proline is one of the more prominent amino acids in the structure of collagen. Proline-containing peptides are involved in a variety of biological processes, such as immune and pro-inflammatory responses (33), as well as in the process of collagen synthesis, which is important for fibroblasts (34). The study found increased proline metabolism, causing dysregulation in arginine and proline metabolism, which may worsen collagen biosynthesis and fibroblast accumulation (35). However, the metabolic level of S-adenosylmethioninamine (SAM) metabolism is downregulated, which is derived from S-adenosylmethionine and acts as a methyl donor in many basic metabolic processes (36). The production of SAM reduces the availability of S-adenosylmethionine (37). This, in turn, affects the level of intracellular polyamines, which influence proline metabolism by affecting the cell cycle and cell death pathways, indirectly affecting proline metabolism (38). Downregulation of the *HMGCS2* gene was found in gene level assays, which may affect ketone body synthesis *in vivo* (39) and inhibit intracellular energy metabolism and redox homeostasis, which in turn affects intracellular NADH/NAD + ratios (40), leading to an impact on arginine and proline metabolism. Also, the regulation of the *HMGCS2* gene involved in energy metabolism under the influence of ketogenic diet or gut microbiota affects butyric acid metabolism (41), which in turn has a regulatory effect on vitamin B6 metabolism.

Through examination of the PPAR signaling pathway, it was noted that lipogenesis is co-regulated by PPAR-*α* and PPAR-*γ*. *FADS2* and *SCD* genes are involved in lipogenesis and the synthesis of unsaturated fatty acids (42). Through regulation of the PPAR signaling pathway, biosynthesis of unsaturated fatty acids, arachidonic acid, and linoleic acid metabolism are influenced. Unsaturated fatty acids significantly protect against PF by affecting PF metabolites and intestinal flora (43). Within the AMPK pathway, the *SCD1* gene catalyzes monounsaturated fatty acid synthesis (44). Upregulation of *SCD1*, *SCD*, and *FADS2* promotes unsaturated fatty acid metabolism, showing a positive role in PF treatment. Activation of the PPAR signaling pathway inhibits pro-inflammatory gene expression (45) and affects arachidonic acid metabolism (46), resulting in anti-inflammatory effects. Leukotriene A4 (*LTA4*) and Prostaglandin F2-alpha (*PGF2α*) are inflammatory mediators derived from the metabolism of arachidonic acid (47), and they play important roles in inflammatory responses. Specifically, *LTA4* is mainly produced via the leukotriene pathway, whereas *PGF2α* is generated via the cyclooxygenase (COX) pathway (48). In the present study, we found that the metabolic levels of *LTA4* and *PGF2α* were markedly downregulated, suggesting that arachidonic acid metabolism, which is regulated via the PPAR signaling pathway, exerts an anti-inflammatory effect. Metabolism exerts an anti-inflammatory effect, thereby enhancing the body’s anti-inflammatory level.

In this study, regulation of the fatty acid degradation pathway was achieved through modulation of the AMPK and PPAR signaling pathways in a rat model of lung fibrosis treated with SRL. Upregulation of the *SCD1* gene in the AMPK pathway catalyzes unsaturated fatty acid synthesis, serving as lipid storage and cellular defense (49). Downregulation of the *LIPE* gene potentially disrupts fatty acid oxidation processes, contributing to monounsaturated fatty acid synthesis and lipid accumulation within cells (50). In the PPAR signaling pathway, *SCD* and *FADS2* play a key role in converting saturated fatty acids to monounsaturated fatty acids (51). Overall, the AMPK and PPAR signaling pathways intricately regulate fatty acid degradation and unsaturated fatty acid biosynthesis, showing significant therapeutic potential in combating oxidative stress, inflammation, endoplasmic reticulum stress, and extracellular matrix deposition in PF (52, 53). Our prior metabolomics investigations into the anti-pulmonary fibrosis effects of SRL have consistently elucidated its regulatory influence on fatty acid metabolism, vitamin B6 metabolism, and associated inflammatory metabolic pathways. These findings offer robust corroborative evidence for the present conclusion that SRL mediates its anti-fibrotic effects through the modulation of PPAR and AMPK signaling pathways. The integration of evidence from both metabolomic and transcriptomic analyses substantially enhances the mechanistic comprehension of SRL’s therapeutic action against pulmonary fibrosis (54).

## 5 Conclusion

In conclusion, SRL demonstrates therapeutic efficacy in bleomycin-induced PF in rats. Integrated analysis of transcriptomics and metabolomics data reveals that SRL alleviates pulmonary fibrosis through modulation of AMPK and PPAR signaling pathways mediated by genes such as *SCD*, *HMGCS2*, *LTA4*, and *FADS2*. These genes play crucial roles in regulating cellular energy metabolism and are closely linked to amino acid and fatty acid metabolic pathways. This study underscores the significance of multi-omics approaches in elucidating the intricate mechanisms of drug action and establishes a scientific foundation for investigating Mongolian pharmaceuticals in the treatment of pulmonary fibrosis. Our formulation demonstrates anti-fibrotic efficacy, indicating its potential clinical applicability for patients with early-stage pulmonary fibrosis. Additionally, the metabolic transcriptome network has been identified as a prospective pharmacodynamic biomarker for future clinical trials, facilitating the monitoring of target engagement. Nevertheless, while the bleomycin model replicates the primary fibrotic characteristics, its self-limiting nature and accelerated timeline may not adequately represent the progression of human pulmonary fibrosis. Future research utilizing alternative models is necessary to enhance predictive accuracy. The current findings primarily address the effects of a single therapeutic intervention. In light of contemporary clinical practices, it is imperative to evaluate the interaction with standard anti-fibrotic medications to position this therapy appropriately. Subsequent investigations will further elucidate the functional roles of these molecules in the pathogenesis of pulmonary fibrosis.

## Data Availability

The datasets presented in this study can be found in online repositories. The names of the repository/repositories and accession number(s) can be found below: https://www.ncbi.nlm.nih.gov/bioproject/PRJNA992181/.

## Footnotes

^1^ https://www.Omicstudio.cn/tool

^2^ https://www.genome.jp/kegg/
